# Preventing *Pseudomonas aeruginosa *and *Chromobacterium violaceum *infections by anti-adhesion-active components of edible seeds

**DOI:** 10.1186/1475-2891-11-10

**Published:** 2012-02-15

**Authors:** Ofra Rachmaninov, Keren D Zinger-Yosovich, Nechama Gilboa-Garber

**Affiliations:** 1 The Mina & Everard Goodman Faculty of Life Sciences, Bar-Ilan University, Ramat-Gan 52900, Israel

**Keywords:** Anti-adhesion activity, Edible seeds, Lectin blocking, *Pseudomonas aeruginosa*, Western blotting

## Abstract

**Background:**

*Pseudomonas aeruginosa *adhesion to animal/human cells for infection establishment involves adhesive proteins, including its galactose- and fucose-binding lectins PA-IL (LecA) and PA-IIL (LecB). The lectin binding to the target-cell receptors may be blocked by compatible glycans that compete with those of the receptors, functioning as anti-adhesion glycodecoys. The anti-adhesion treatment is of the utmost importance for abrogating devastating antibiotic-resistant *P. aeruginosa *infections in immunodeficient and cystic fibrosis (CF) patients. This strategy functions in nature in protecting embryos and neonates. We have shown that PA-IL, PA-IIL, and also CV-IIL (a PA-IIL homolog produced in the related pathogen *Chromobacterium violaceum*) are highly useful for revealing natural glycodecoys that surround embryos in diverse avian eggs and are supplied to neonates in milks and royal jelly. In the present study, these lectins were used as probes to search for seed embryo-protecting glycodecoys.

**Methods:**

The lectin-blocking glycodecoy activities were shown by the hemagglutination-inhibition test. Lectin-binding glycoproteins were detected by Western blotting with peroxidase-labeled lectins.

**Results:**

The present work reports the finding - by using PA-IL, PA-IIL, and CV-IIL - of rich glycodecoy activities of low (< 10 KDa) and high MW (> 10 kDa) compounds (including glycoproteins) in extracts of cashew, cocoa, coffee, pumpkin, and tomato seeds, resembling those of avian egg whites, mammal milks, and royal jelly.

**Conclusions:**

Edible seed extracts possess lectin-blocking glycodecoys that might protect their embryos from infections and also might be useful for hampering human and animal infections.

## Introduction

The worldwide-distributed *Pseudomonas aeruginosa *and the tropical-subtropical *Chromobacterium violaceum *are soil saprophytic bacteria that are occasionally transformed into opportunistic aggressive animal (including human) pathogens [1,2]. They adhere to target cells and to each other by means of diverse adhesins, including hemagglutinating carbohydrate-specific lectins [3]. *P. aeruginosa *produces a galactophilic lectin PA-IL (LecA) and a fucophilic (+ mannophilic and arabinophilic) lectin PA-IIL (LecB) [3]. *C. violaceum *also possesses a fucophilic lectin CV-IIL homologous to PA-IIL in structure and major specificity [4]. These three lectins bind to most human cells due to their affinities to their most common antigens [5,6]: PA-IL preferentially binds to the terminal Galα-bearing human blood group epitopes found in P-system, I, and B antigens [5]. PA-IIL binds to both Fucα1-2-bearing H antigen and Fucα1-3/4-bearing Lewis antigens (displaying outstandingly high preferential Le^a ^affinity [7,8]), and also exhibits high affinity to branched oligomannosides. CV-IIL is more selective, preferentially binding to the Fucα1-2-bearing H antigen [4].

These lectins themselves not only bind to, but also affect the target cells and augment the notorious effects of the other bacterial virulence factors, amplifying the host-cell damage [5-7,9]. Since *P. aeruginosa *infections have become resistant to antibiotic treatment, the alterative strategy of hampering its adhesion by means of glycosylated compounds that attract its lectins is of the utmost importance, and the search for compatible glycodecoys has become a major goal [9-11].

PA-IL binding to cell receptors is inhibitable by D-galactose (Gal) and L-arabinose (Ara), while PA-IIL and CV-IIL bindings are blocked by L-fucose (Fuc)-/D-mannose (Man)-/D -arabinose (Ara) [3,4,6,7,10]. Oligomeric complexes of these sugars, which are much stronger inhibitors of PA-IL and PA-IIL binding than monosaccharides [7,8], are also much more efficient in blocking bacterial adhesion [12].

In nature, there is an abundance of *P. aeruginosa *patholectin-blocking glycodecoys that help to protect animal embryos and neonates from its infections. Using PA-IL, PA-IIL, and CV-IIL as probes enabled us to identify such compounds in avian egg whites [13], in human and various other mammalian milks [14,15], in honey, and in royal jelly [16]. Human milk is superior for PA-IIL blocking due to its Le^a ^epitope content [14,15].

Recently, the widely used commercial food additives E-410 and E-412, which are galactomannans of the leguminous locust (carob, *Ceratonia siliqua*) and guar (*Cyamopsis tetragonoloba*) seeds, were also shown to very strongly block PA-IL [17]. These galactomannans (consisting of Man β1-4-linked backbone [scaffold] bearing Galα1-6-branches, with Gal:Man ratios of 1:3.5-4.0 and 1:1.5-2.0, respectively) are present in these leguminous seeds at high levels (accounting for 35-40% of carob seed mass). By blocking PA-IL, they may also contribute to the protection of their embryos against infections [17], similar to the protection provided to avian embryos by their egg-white glycans [13].

The above findings led us to examine the anti-adhesive efficiency of several edible seed extracts, including cashew (*Anacardium occidentale*), cocoa (*Theobroma cacao*), coffee (*Coffee arabica*), pumpkin (*Curcubita maxima*), and tomato (*Lycopersicon esculentum*). This was accomplished using PA-IL, PA-IIL, and CV-IIL lectins as probes. The blocking of the lectins by the glycodecoys was followed by hemagglutination inhibition (HAI). Lectin-binding glycoproteins (GPs) were detected by Western blotting (Wb).

## Methods

### Lectin Preparations

The bacterial lectins PA-IL, PA-IIL, and CV-IIL were purified from cell extracts of *P. aeruginosa *ATCC 33347, and *C. violaceum *(Bergonzini) ATCC 12472 respectively, which were purchased from the American Type Culture Collection (ATCC) (Manassas, VA), as earlier described [3,4]. The purified lectin qualities were controlled by SDS-PAGE with Coomassie brilliant blue staining.

### The Seed Extract Preparations

Fresh seed extracts of cashew (*Anacardium occidentale*), cocoa (*Theobroma cacao*), coffee (*Coffee arabica*), pumpkin (*Cucurbita maxima*), and tomato (*Lycopersicon esculentum*), were ground using a coffee grinder and their powders were suspended (10% W/V) in PBS (0.025 M phosphate-buffered saline [0.85% NaCl] at pH 7.2) with overnight stirring at 4°C. Each suspension was then centrifuged (10,000 xg) for 10 min and the supernatant fluid was carefully collected. A half-volume of each supernatant was dialyzed (using dialysis membrane cut-off of 10 kD) against PBS (at pH 7.2). Both the nondialyzed and dialyzed preparations were divided into several 1-ml aliquots for storage at -20°C.

### Hemagglutinating Activity (HA) and its Inhibition (HAI) Tests

Papain-treated human type O(H) red blood cells (erythrocytes, kindly obtained from the Magen David Adom National Blood Services in Israel) were used. They were prepared by three washings of the cells with PBS (at pH 7.2), followed by their treatment by 0.1% papain with 0.01% cysteine, as previously described [3]. A 50-μl sample of each bacterial lectin preparation examined (at 1 mg/ml concentration) was serially diluted in tubes with 50 μl saline to produce twofold dilutions. After that, saline and 5% (V/V) erythrocyte suspension in saline (50 μl each) were added to each tube. After 30 min at room temperature, the tubes were centrifuged for 30 sec (1000 xg), and hemagglutinating activity was examined, as previously described [3]. HA was represented by the number of twofold dilutions in which there was visible hemagglutination (e.g., Log _2 _dilution^-1 ^= 7 means positive reaction up to dilution of 1:128, or original activity of 128 hemagglutination units).

In the HAI test, each examined seed extract was serially twofold diluted in 50 μl saline, and then 50 μl of the lectin solution (at highest dilution leading to agglutination of all the erythrocytes in one large mass) was added to each tube. After 30 min at room temperature, 50 μl of the 5% papain-treated human O blood-type erythrocyte suspension was added to each tube and after another 30 min, hemagglutination was examined as described above [3]. HAI intensity was represented by the number of twofold dilutions (log _2 _dilution^-1^) without considerable hemagglutination preceding its reappearance.

### Western Blot (Wb) Analyses

The Wb analyses demonstrate the differential lectin interactions with individual, electrophoretically (SDS-PAGE) separated glycoproteins (GPs). Fifteen-μl samples of each examined preparation at a concentration of around 1 mg/ml were used for this test so that both the discrete GP bands and the intensities of their staining by the lectins would be represented. These samples were mixed 1:1 with sample buffer, boiled, and applied to the wells in 10% SDS-PAGE (at 140 V) in Mini-PROTEAN Cell 3 Electrophoresis (Bio-Rad), as previously described. Following SDS-PAGE, the proteins were transferred to nitrocellulose (0.45 μm, Bio-Rad) membrane at 4°C for 2 h (85 mA/40-50 V) using the Mini Trans-Blot Module (Bio-Rad, Haifa, Israel). The membranes were incubated overnight in blocking buffer (PBS 0.02 M, pH 7.2, containing 3% bovine serum albumin [BSA] and 0.05% Tween 20), exposed to horseradish-peroxidase-labeled lectins (about 1 μg ml^-1^, dissolved in the same blocking buffer with 0.1% Tween 20) at room temperature for 2 h, and then thoroughly washed. The peroxidase reaction was visualized using enhanced chemiluminescence (Amersham International PLC, Buckinghamshire, UK) and recorded onto photographic films. Controls with the peroxidase-labeled lectins in the presence of 0.3 M of the relevant blocking sugars in their reaction mixtures were used in parallel in order to rule out nonspecific (sugar-independent) lectin binding. Positive controls were those with human milk, quail egg white, royal jelly, and honey, which strongly block these lectins.

### Statistical Evaluation

**Figure 1:**
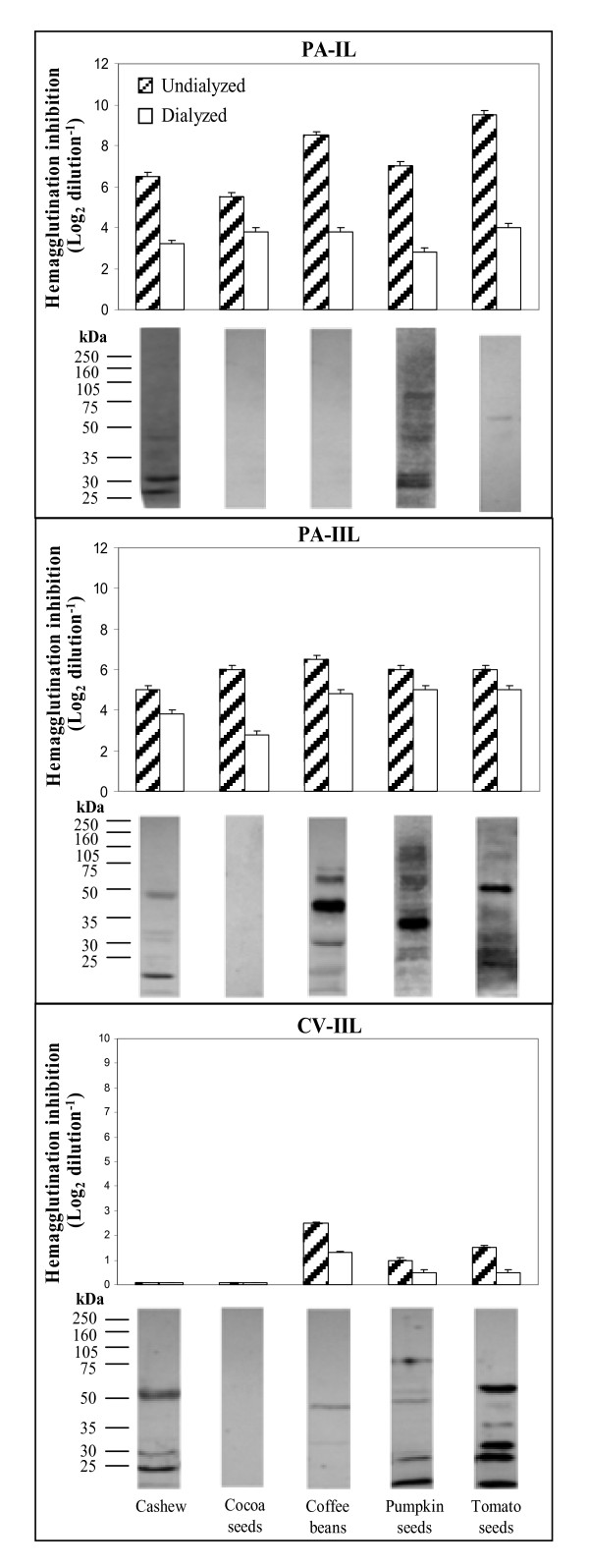
**Interactions (hemagglutination inhibitions and Western blots) of the seed extracts with PA-IL, PA-IIL, and CV-IIL**.

**Figure 2:**
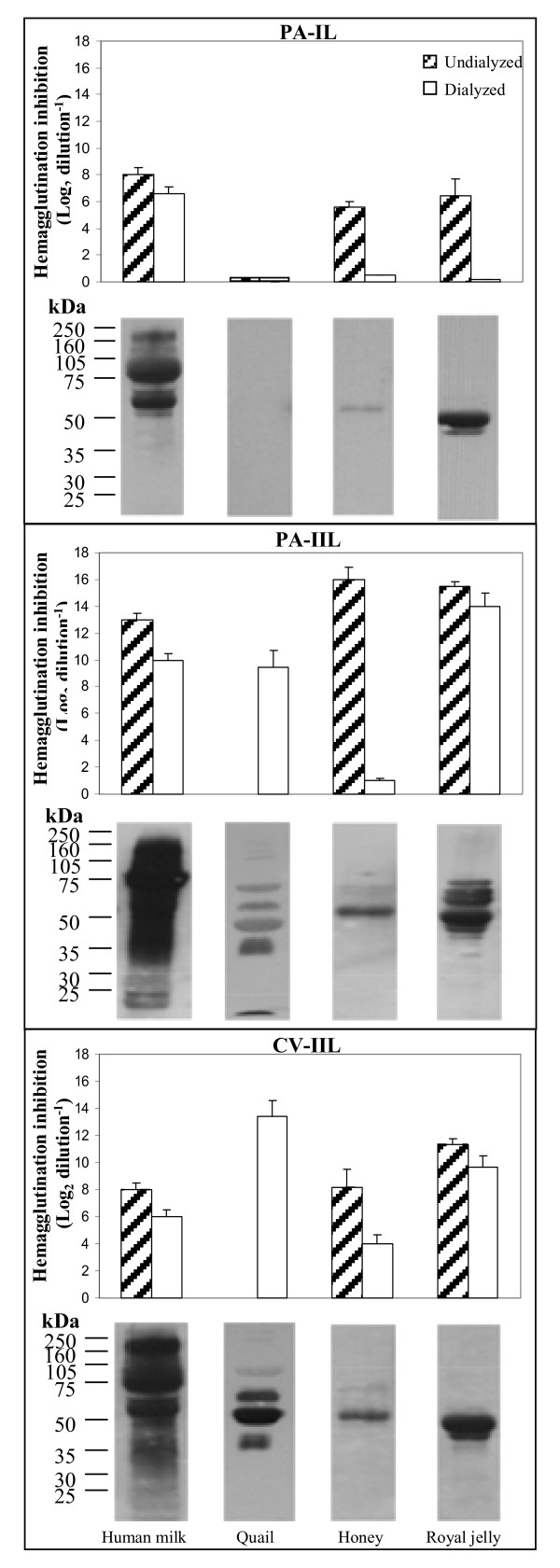
**PA-IL, PA-IIL, and CV-IIL interactions with human milk, quail egg white, honey, and royal jelly**.

The results of hemagglutination and its inhibition tests were analyzed by Student's *t*-test. The data presented in Figures 1 and 2 represent means + SEM of at least five experimental results for each lectin.

## Results and Discussion

**Figure 3:**
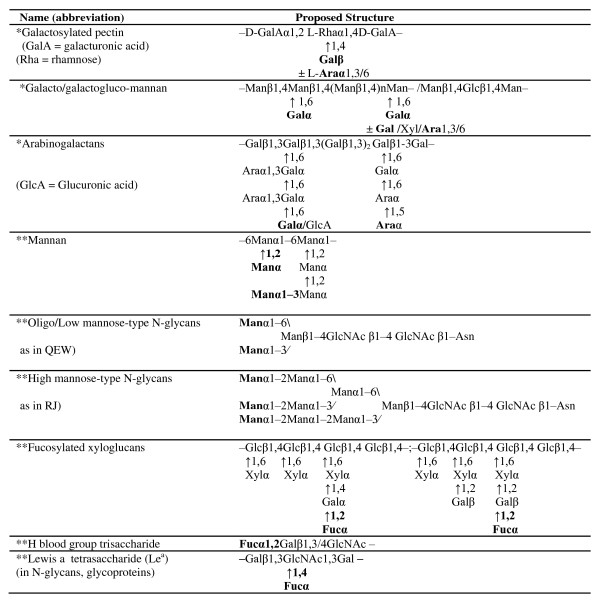
**The possible structures of the active lectin-binding epitopes of the examined seed galactosylated (PA-IL-binding), mannosylated, and fucosylated (PA-IIL-binding and CV-IIL-binding) oligosaccharides**. Binding sugars are marked in bold

Seeds contain high levels of polysaccharides (PSs), which function as substrate reserves for germination and as osmoprotectants [18]. In addition, they may also contribute to the protection of embryos against infections by blocking pathogenic bacterial lectins [17]. The differential interactions of the five seed extracts with the patholectins PA-IL, PA-IIL, and CV-IIL, are compiled in Figure 1 and Tables 1 and 2. The herein-examined seeds of cashew, cocoa, coffee, pumpkin, and tomato were chosen due to their edibility and their rich nonstarch PS reserves, which were thoroughly studied by experts in the field [18-24]. In general, most seed PSs contain galactans, galactomannans, mannans, and xyloglucans (Table 3). Among the examined seeds, those of cocoa beans were reported to be outstanding in their major, highly branched, pectic PSs (60% of the total cell-wall PSs), constructed of rhamnogalacturonan backbone heavily substituted by 5-linked Ara and 4-linked Gal side chains [19]. In addition, they were found to contain fucosylated xyloglucan (Figure 3) and galactoglucomannan [19] consisting of 1-4-linked Glc and Man backbone, with 42% of the Man residues and 13% of the Glc residues substituted at O-6 by Gal or by several Gal pairs, with either Gal, Ara, or xylose as the terminal saccharide [19]. The coffee-bean cell-wall PSs, which constitute half the bean dry weight [20], contain galactomannans composed of 1-4-linked β Man backbone substituted at O-6 by single Gal residues (Table 3) and type-II arabinogalactans (consisting of a 1-3-linked β-Gal backbone substituted mainly at O-6 by side chains of Gal, Ara, and Rha residues and 2 mol % of glucuronic acid residues with the Ara as the terminal residue). The latter are usually covalently linked to proteins containing 10% of 4-hydroxyproline residues [20,21]. The pumpkin seeds were found to contain high mannose-type free N-glycans [22] (Table 3) and the tomato seeds contain approximately 60% Man, largely as β1-4-mannan backbone, with lesser amounts of glucose, Gal, and Ara, probably in the form of α-Gal side chain-bearing galactomannans or galactoglucomannans [23], which decompose during germination [24]. The blocking of the *P. aeruginosa *and *C. violaceum *lectins by the examined seed extracts (Figure 1 and Table 1) was compatible with the above-described composition of their glycans. The staining of the seed-extract Wbs by the peroxidase-labeled bacterial lectins has added important novel information as to their epitope-bearing GPs (Figure 3 and Table 3).

**Table 1:** Patholectin-inhibiting glycodecoy activities in non-dialyzed (N) and dialyzed (D) seed extract preparations

	Preparation and test																	
	
	L/G	A	M	Cashew			Cocoa			Coffee			Pumpkin			Tomato		
	
Lectin	HAI	HAI	HAI	HAI		Wb	HAI		Wb	HAI		Wb	HAI		Wb	HAI		Wb
				N	D	Bn	N	D	Bn	N	D	Bn	N	D	Bn	N	D	Bn
PA-IL	35/20	13	<1	6.5	3	3	5.5	4	0	8.5	4	vw	7	3	4	9	4	vw

PA-IIL	0/0	2	16	5	4	3	6	3	0	6.5	5	5	6	5	7	6	5	7

CV-IIL	0/0	3	1	<1	<1	4	<1	<1	0	2.5	1.5	3w	1	0.5	4	1.5	0.5	6

Lectin-inhibiting activities are expressed by numbers representing their hemagglutination titer reduction (Log_2 _dilution^-1^) by the five edible seed extracts, as compared to those of locust (L) and guar (G) bean gum galactomannans, *Acacia arabic *gum (A), and mannan (M) [17]. The data include results of hemagglutination inhibition tests (HAI) and counts of the observed GP bands (Bn) in the lectin-stained western blots (Wb) of these seeds, excluding the very weak (vw) ones.

**Table 2:** Comparison of the edible seed glycodecoy inhibitory activities to those of animal embryo-protecting and neonate-protecting substances

	Preparation and test																			
	
	HM			CM			QEW		RJ			Honey			Pumpkin			Tomato		
	
Lectin	HAI		Wb	HAI		Wb	HAI	Wb	HAI		Wb	HAI		Wb	HAI		Wb	HAI		Wb
	N	D	Bn	N	D	Bn	D	Bn	N	D	Bn	N	D	Bn	N	D	Bn	N	D	Bn
PA-IL	8	6.5	5	1	<1	0	<1	0	6	<1	3	5	<1	1w	7	3	4	9	4	vw

PA-IIL	13	10	20	<1	<1	1	9	>5	16	14	10	16.5	1	3	6	5	7	6	5	7

CV-IIL	8	6	>15	1	1	0	13	>5	11	10	3	8	3.5	2	1	0.5	4	1.5	0.5	6

The lectin-inhibiting activities are expressed in numbers representing the hemagglutination titer reduction (Log_2 _dilution^-1^) of the three patholectins by non-dialyzed (N) and dialyzed (D) preparations of pumpkin seed and tomato seed extracts, as compared to animal embryo-protecting and neonate-protecting substances (human milk (HM), cow milk (CM), quail egg white (QEW), royal jelly (RJ), and honey [16]). The data include the results of hemagglutination inhibition tests (HAI) and counts of the observed GP bands (Bn) in these lectin-stained western blots (Wb), excluding the very weak (vw) ones.

**Table 3:** The active seed glycodecoy epitopes involved in the blocking of PA-IL, PA-IIL, and CV-IIL (graded on an intensity scale of ± - ++++)

Epitope	[terminal sugar]	PA-IL	PA-IIL	CV-IIL
Galactomannan	[Galα1,6]	++++	-	-
Arabinogalactan	[Galα1,6]	++	-	-
	[Araα1,5]	++	+	+
Fucosylxyloglucan (XXFG)	[Fucα1,2]	-	++	++
H blood group	[Fucα1,2]	-	+++	++ +
Lewis a (Le^a^)	[Fucα1,4]	-	++++	±

Figure 1 and Table 1 show that *P. aeruginosa *Gal-binding PA-IL, which was most sensitive to blocking by the locust- and guar-bean galactomannans [17], was also inhibited by the cashew-, cocoa-, coffee-, pumpkin-, and tomato-seed extracts, as expected based on the documented presence of Gal-bearing glycans in all of them (Figure 3 and Table 3). The significant inhibition of PA-IL by the cocoa-seed extract is compatible with the description of the special Gal-bearing, highly branched pectic PSs and galactoglucomannans in the seeds [19]. The highest inhibitions of PA-IL by the coffee- and tomato-seed extracts are also in line with the reports on the coffee Gal-bearing galactomannans and arabinogalactans [20] and the tomato galactomannans and galactoglucomannans [23].

Comparison of nondialyzed (containing both low MW [LMW, < 10 kDa] and high MW [HMW] glycans) to dialyzed seed extracts (retaining only the HMW [> 10 kDa] glycans) revealed that PA-IL blocking was due to both LMW and HMW saccharides (Figure 1). The PA-IL-stained Wbs of the tomato seed extracts revealed one pale GP band (at around 55-60 kDa). In the cashew Wb there were 2 strong GP bands (at around 27 and 31 kDa) and a few weaker ones (at 33, 37, and 45 kDa). In the pumpkin, there were more than 10 bands between 28 and 160 kDa, with those around 30, 31, and 80 kDa being the boldest.

Interestingly, most of the PA-IL-stained cashew and pumpkin GP bands and the one tomato band were also stained by PA-IIL and CV-IIL, revealing that their GP oligosaccharides were of hybrid type, with both Gal- and Fuc- or Man-type bearing antennae.

Figure 1 shows that *P. aeruginosa *fucophilic (Fuc-, Man-, and D-Ara-binding) lectin PA-IIL was inhibited by the same five seed extracts. Its inhibition was not due to the PA-IL-blocking galactomannans (Tables 1 and Figure 3) [17], but might be due either to terminal Fucα1-2-linked residues carried on β-Gal side chains of some xyloglucans and arabinogalactans [19] or to high-mannose-type N-glycans in either free form (e.g., in the pumpkin seeds) [22] or linked to macromolecules. The PA-IIL-binding GP bands might contain either oligo or high-mannose-type N-glycans or Le^a^-epitope (Figure 3), which is the best PA-IIL ligand [8,9] that also contributes to its highest blocking by human milk (Figures 2, 3 and Tables 2 and 3) [14,15,25]. Le^a^- bearing antennae are found in plants as short N-glycans and in association with PSs and GPs [26-28]. PA-IIL staining of the examined-seed Wbs exhibited considerably more GP bands than PA-IL: at least 10 each in the coffee, pumpkin, and tomato lanes. The PA-IIL-stained pumpkin-seed Wb displayed around 15 GP bands, most of them also stained by PA-IL. However, the relative intensities of the staining of these bands by the 2 lectins were not similar: while PA-IL most strongly stained the bands at around 25-30 kDa, PA-IIL most strongly stained a band at around 33 kDa.

As seen in Figure 1, in contrast to the two *P. aeruginosa *lectins, the *C. violaceum *fucophilic lectin CV-IIL displayed low sensitivity to the coffee-, tomato- and pumpkin-seed glycans and was negligibly inhibited by the cashew- and cocoa-seed extracts. Three GP bands were observed in the CV-IIL-stained cashew Wb. They also interacted with PA-IL and PA-IIL. The CV-IIL staining of the 50-kDa-cashew GP was darker than that observed with PA-IIL (this band was not stained by PA-IL). The CV-IIL-stained coffee and pumpkin-seed Wbs showed weaker interactions, while in the tomato-seed Wb there were four bold bands (at around 10, 30, 32, and 60 kDa) and 2 weaker ones (at 35 and 40 kDa), all of them also seen in the respective PA-IIL-stained Wbs. Weak CV-IIL inhibitions by the seed extracts (as opposed to PA-IIL and its own blocking by the animal products [Figure 2 and Table 2]), can be ascribed to the strict selectivity of this lectin (also exhibited in its insensitivity to inhibition by the PA-IIL-blocking yeast mannan) (Tables 1 and 3). The GP bands observed in the CV-IIL-stained cashew, pumpkin, and especially tomato Wbs were not associated with considerable lectin blocking, probably due to either the low level of the GPs or low affinity to them. Lack of correlation between lectin-binding intensity and Wb-band staining is not surprising since there is generally no quantitative correlation between the intensities of these two parameters.

The shared PA-IIL and CV-IIL bands in the cashew-, pumpkin-, and tomato-seed Wbs might represent the Fucα1-2 residues linked to GPs through asparagine-bound N-glycans, as described by Puhlmann et al. [29]. The exclusive PA-IIL-stained coffee and pumpkin GP bands, not stained by CV-IIL, confirm the higher selectivity of the latter (Table 3).

## Conclusions

The usage of PA-IL, PA-IIL, and CV-IIL patholectins as probes has revealed rich glycodecoy arsenals in the edible cashew, cocoa, coffee, pumpkin and tomato seeds. These lectin-blocking activities, which are in accord with the reports on the composition of these seed polysaccharides, show their important potential contribution to the protection of embryos against infections. PA-IL blocking by these seed glycans was even more efficient than by human milk and royal jelly. PA-IIL was also nicely inhibited by the five seed-extract glycans (albeit much less than by human milk and royal jelly). CV-IIL, which was weakly inhibited by the seed extracts, did stain several GPs. Based on the herein-presented data, the examined seed glycans might be considered as efficient clinical agents for reducing intestinal and external animal and human infections by blocking lectin-dependent bacterial adhesion. The only warning that should be issued is about possible allergic reactions, such as that known to be caused by cashew seeds [30].

## List of Nonstandard Abbreviations

**Ara: **arabinofuranose; ***C. violaceum***: *Chromobacterium violaceum***; CV-IIL: ***C. violaceum *lectin; **Fuc: **L-fucose; **Gal: **D-galactopyranose; **GP: **glycoprotein; **HA: **hemagglutinating activity; **HAI: **hemagglutinating activity inhibition; **HM: **human milk; **HMW**: high molecular weight; **Le^a^**: Lewis a; **Man**: D-mannose; ***P. aeruginosa***: *Pseudomonas aeruginosa*; **LMW**: low molecular weight; **PA-IL (**LecA): *P. aeruginosa *first (galactophilic) lectin; **PA-IIL (**LecB): *P. aeruginosa *second (fucophilic) lectin; **PBS**: phosphate-buffered (0.025 M: pH 7.2) saline (isotonic 0.85% NaCl solution); **PS: **polysaccharide; **Rha **L-rhamnopyranose **RJ: **royal jelly; **Wb: **Western blotting.

## Competing interests

The authors declare that they have no competing interests.

## Authors' contributions

This manuscript is part of the PhD thesis of OR, who conducted the research under the supervision of NGG. KDZY helped her in the technical work. All authors read and approved the final version of the manuscript.
